# Supplementary material for: FastqPuri: high-performance preprocessing of RNA-seq data
Source: BMC Bioinformatics. 2019 May 3;20:226. doi: 10.1186/s12859-019-2799-0 (PMC6500068; doi:10.1186/s12859-019-2799-0)
Supplement: Supplementary file 2 — Archive of FastqPuri. Archive containing all files needed to install and run FastqPuri v1.0.6. Date stamp March 22, 2019. (GZ 47,819 kb) [file 12859_2019_2799_MOESM2_ESM.gz › FastqPuri-1.0.6/examples/Qreport_Sreport/test_output.html]

Assessing the quality of the reads


# Assessing the quality of the reads

## General information

Running on version 1.0.3

| Var | Value |
| --- | --- |
| Input file name | test\_output.bin |
| Read length | 51 |
| Min good quality | 27 |
| Number of reads | 500000 |
| Number of highQ reads | 336682 |
| Number of tiles | 20 |
| Number of lanes | 2 |
| Qualities | 2 (#), 14 (/), 22 (7), 27 (<), 33 (B), 37 (F) |
| Reads with N’s | 812 |
| Number of N’s | 1114 |

## Per base sequence quality

## # reads with at least `m` low Q nucleotides

## Low Q nucleotide proportion per tile per lane

## Average quality per position per tile per lane

## Low Q nucleotides proportion per position per tile per lane

## Low Q nucleotides proportion per position for all tiles

## Nucleotide content per position
