# Supplementary material for: FastqPuri: high-performance preprocessing of RNA-seq data
Source: BMC Bioinformatics. 2019 May 3;20:226. doi: 10.1186/s12859-019-2799-0 (PMC6500068; doi:10.1186/s12859-019-2799-0)
Supplement: Supplementary file 2 — Archive of FastqPuri. Archive containing all files needed to install and run FastqPuri v1.0.6. Date stamp March 22, 2019. (GZ 47,819 kb) [file 12859_2019_2799_MOESM2_ESM.gz › FastqPuri-1.0.6/examples/Qreport_Sreport/test_summary_report.html]

Summary quality report


# Summary quality report

Running on version 1.0.3

## General data

|  | # reads | # tiles | % lowQ reads | % reads with N’s |
| --- | --- | --- | --- | --- |
| test\_output | 5e+05 | 20 | 32.6636 | 0.1624 |

## Mean quality
