# Supplementary material for: FastqPuri: high-performance preprocessing of RNA-seq data
Source: BMC Bioinformatics. 2019 May 3;20:226. doi: 10.1186/s12859-019-2799-0 (PMC6500068; doi:10.1186/s12859-019-2799-0)
Supplement: Supplementary file 2 — Archive of FastqPuri. Archive containing all files needed to install and run FastqPuri v1.0.6. Date stamp March 22, 2019. (GZ 47,819 kb) [file 12859_2019_2799_MOESM2_ESM.gz › FastqPuri-1.0.6/html/Lmer_8c.html]

FastqPuri: src/Lmer.c File Reference


|  |
| --- |
| FastqPuri |


- src

Functions |
Variables

Lmer.c File Reference

Manipulation of Lmers and sequences.
More...

`#include "Lmer.h"`  
`#include <string.h>`  
`#include <stdint.h>`  
`#include <stdio.h>`

Include dependency graph for Lmer.c:

|  |  |
| --- | --- |
| Functions | |
| void | init\_map () |
|  | Initialize lookup table fw\_1B. More... |
|  | |
| void | Lmer\_sLmer (char \*Lmer, int L) |
|  | Transforms an Lmer to the convention stored in the lookup table fw\_1B. |
|  | |
| void | rev\_comp (char \*sLmer, int L) |
|  | Obtains the reverse complement, for {'\000','\001','\002','\003'}. |
|  | |

|  |  |
| --- | --- |
| Variables | |
| uint8\_t | fw\_1B [256] |
|  | |
| uint8\_t | bw\_1B [256] |
|  | |
| uint8\_t | Nencode |
|  | |

## Detailed Description

Manipulation of Lmers and sequences.

Author
:   Paula Perez paula.nosp@m.pere.nosp@m.zrubi.nosp@m.o@gm.nosp@m.ail.c.nosp@m.om

Date
:   18.08.2017

## Function Documentation

## ◆ init\_map()

|  |  |  |  |  |
| --- | --- | --- | --- | --- |
| void init\_map | ( |  | ) |  |

Initialize lookup table fw\_1B.

{'a','c','g','t'} –> {'\000','\001','\002','\003'}, rest '\004'.

## Variable Documentation

## ◆ bw\_1B

|  |
| --- |
| uint8\_t bw\_1B[256] |

global variable. Lookup table.

## ◆ fw\_1B

|  |
| --- |
| uint8\_t fw\_1B[256] |

global variable. Lookup table.

## ◆ Nencode

|  |
| --- |
| uint8\_t Nencode |

global variable. Encoding for N's(\004)


---

Generated on Mon Mar 19 2018 23:42:01 for FastqPuri by  

 1.8.14
