# Supplementary material for: FastqPuri: high-performance preprocessing of RNA-seq data
Source: BMC Bioinformatics. 2019 May 3;20:226. doi: 10.1186/s12859-019-2799-0 (PMC6500068; doi:10.1186/s12859-019-2799-0)
Supplement: Supplementary file 2 — Archive of FastqPuri. Archive containing all files needed to install and run FastqPuri v1.0.6. Date stamp March 22, 2019. (GZ 47,819 kb) [file 12859_2019_2799_MOESM2_ESM.gz › FastqPuri-1.0.6/html/Rcommand__Qreport_8h.html]

FastqPuri: include/Rcommand\_Qreport.h File Reference


|  |
| --- |
| FastqPuri |


- include

Functions

Rcommand\_Qreport.h File Reference

get Rscript command for Qreport
More...

This graph shows which files directly or indirectly include this file:

Go to the source code of this file.

|  |  |
| --- | --- |
| Functions | |
| char \* | command\_Qreport () |
|  | returns Rscript command that generates the quality report in html |
|  | |

## Detailed Description

get Rscript command for Qreport

Author
:   Paula Perez paula.nosp@m.pere.nosp@m.zrubi.nosp@m.o@gm.nosp@m.ail.c.nosp@m.om

Date
:   09.08.2017


---

Generated on Mon Mar 19 2018 23:42:01 for FastqPuri by  

 1.8.14
