# Supplementary material for: FastqPuri: high-performance preprocessing of RNA-seq data
Source: BMC Bioinformatics. 2019 May 3;20:226. doi: 10.1186/s12859-019-2799-0 (PMC6500068; doi:10.1186/s12859-019-2799-0)
Supplement: Supplementary file 2 — Archive of FastqPuri. Archive containing all files needed to install and run FastqPuri v1.0.6. Date stamp March 22, 2019. (GZ 47,819 kb) [file 12859_2019_2799_MOESM2_ESM.gz › FastqPuri-1.0.6/html/Rcommand__Sreport_8h.html]

FastqPuri: include/Rcommand\_Sreport.h File Reference


|  |
| --- |
| FastqPuri |


- include

Functions

Rcommand\_Sreport.h File Reference

get Rscript command for Sreport
More...

This graph shows which files directly or indirectly include this file:

Go to the source code of this file.

|  |  |
| --- | --- |
| Functions | |
| char \* | command\_Sreport () |
|  | returns Rscript command that generates the summary report in html More... |
|  | |

## Detailed Description

get Rscript command for Sreport

Author
:   Paula Perez paula.nosp@m.pere.nosp@m.zrubi.nosp@m.o@gm.nosp@m.ail.c.nosp@m.om

Date
:   09.08.2017

## Function Documentation

## ◆ command\_Sreport()

|  |  |  |  |  |
| --- | --- | --- | --- | --- |
| char\* command\_Sreport | ( |  | ) |  |

returns Rscript command that generates the summary report in html

# To run between quotation marks after: Rscript\_RBioC -e (Rscript)

inputfolder = normalizePath( <par.SR.inputfolder>, mustWork = TRUE);

output = <par\_SR.outputfile>;

output\_file = gsub('.\* /', '', output);

path = gsub('[^/]+$', '', output);

if (path != '') {

outputfile = paste0(normalizePath(path, mustWork = TRUE), '/', outputfile);

} else {

outputfile = paste0(cwd, '/', output\_file); # cwd: current working dir

};

rmarkdown::render(<par\_SR.Rmd\_file>,

params = list(inputfolder = inputfolder, version= VERSION),

output\_file = output\_file)


---

Generated on Mon Mar 19 2018 23:42:01 for FastqPuri by  

 1.8.14
