# Supplementary material for: FastqPuri: high-performance preprocessing of RNA-seq data
Source: BMC Bioinformatics. 2019 May 3;20:226. doi: 10.1186/s12859-019-2799-0 (PMC6500068; doi:10.1186/s12859-019-2799-0)
Supplement: Supplementary file 2 — Archive of FastqPuri. Archive containing all files needed to install and run FastqPuri v1.0.6. Date stamp March 22, 2019. (GZ 47,819 kb) [file 12859_2019_2799_MOESM2_ESM.gz › FastqPuri-1.0.6/html/Sreport_8c.html]

FastqPuri: src/Sreport.c File Reference


|  |
| --- |
| FastqPuri |


- src

Functions |
Variables

Sreport.c File Reference

Sreport main function.
More...

`#include <stdio.h>`  
`#include <stdlib.h>`  
`#include <time.h>`  
`#include "init_Sreport.h"`  
`#include "Rcommand_Sreport.h"`  
`#include "config.h"`

Include dependency graph for Sreport.c:

|  |  |
| --- | --- |
| Functions | |
| int | main (int argc, char \*argv[]) |
|  | Qreport main function. |
|  | |

|  |  |
| --- | --- |
| Variables | |
| Iparam\_Sreport | par\_SR |
|  | |

## Detailed Description

Sreport main function.

Author
:   Paula Perez paula.nosp@m.pere.nosp@m.zrubi.nosp@m.o@gm.nosp@m.ail.c.nosp@m.om

Date
:   09.08.2017 This file contains the summary report main function. Given a folder containing \*bin as from Qreport output, Sreport generates a summary report in html format. See README\_Sreport.md for more details.

## Variable Documentation

## ◆ par\_SR

|  |
| --- |
| Iparam\_Sreport par\_SR |

input parameters Sreport


---

Generated on Mon Mar 19 2018 23:42:01 for FastqPuri by  

 1.8.14
