# Supplementary material for: FastqPuri: high-performance preprocessing of RNA-seq data
Source: BMC Bioinformatics. 2019 May 3;20:226. doi: 10.1186/s12859-019-2799-0 (PMC6500068; doi:10.1186/s12859-019-2799-0)
Supplement: Supplementary file 2 — Archive of FastqPuri. Archive containing all files needed to install and run FastqPuri v1.0.6. Date stamp March 22, 2019. (GZ 47,819 kb) [file 12859_2019_2799_MOESM2_ESM.gz › FastqPuri-1.0.6/html/annotated.html]

FastqPuri: Class List


|  |
| --- |
| FastqPuri |


Class List

Here are the classes, structs, unions and interfaces with brief descriptions:

|  |  |
| --- | --- |
| C\_ad\_seq | Stores an adapter entry |
| C\_adapter |  |
| C\_bfilter | Bloom filter structure |
| C\_bfkmer | Stores a processed kmer (2 bits pro nucleotide) |
| C\_ds\_adap | Structure containing an adapter pair (for read 1 and read 2) |
| C\_fa\_data | Stores sequences of a fasta file |
| C\_fa\_entry | Fasta entry |
| C\_fq\_read | Stores a fastq entry |
| C\_iparam\_makeBloom | MakeBloom input parameters |
| C\_iparam\_makeTree | MakeTree input parameters |
| C\_iparam\_Qreport | Qreport input parameters |
| C\_iparam\_Sreport | Sreport input parameters |
| C\_iparam\_trimFilter | TrimFilter input parameters |
| C\_node | Node structure: formed out of T\_ACGT pointers to Node structure |
| C\_split | Splitted string and the number or splitted fields |
| C\_stats\_TF | Collects stats info from the filtering procedure |
| C\_stats\_TFDS | Collects stats info from the filtering procedure |
| C\_tree | Structure containing a T\_ACGT-tree |
| C\_uint128 |  |
| Cstatsinfo | Stores info needed to create the summary graphs |


---

Generated on Mon Mar 19 2018 23:42:01 for FastqPuri by  

 1.8.14
