# Supplementary material for: FastqPuri: high-performance preprocessing of RNA-seq data
Source: BMC Bioinformatics. 2019 May 3;20:226. doi: 10.1186/s12859-019-2799-0 (PMC6500068; doi:10.1186/s12859-019-2799-0)
Supplement: Supplementary file 2 — Archive of FastqPuri. Archive containing all files needed to install and run FastqPuri v1.0.6. Date stamp March 22, 2019. (GZ 47,819 kb) [file 12859_2019_2799_MOESM2_ESM.gz › FastqPuri-1.0.6/html/city_8h.html]

FastqPuri: include/city.h File Reference


|  |
| --- |
| FastqPuri |


- include

Classes |
Macros |
Typedefs |
Functions

city.h File Reference

functions for hashin strings, C translation of cityhash (C++, google)
More...

`#include <stdlib.h>`  
`#include <stdint.h>`

Include dependency graph for city.h:

This graph shows which files directly or indirectly include this file:

Go to the source code of this file.

|  |  |
| --- | --- |
| Classes | |
| struct | \_uint128 |
|  | |

|  |  |
| --- | --- |
| Macros | |
| #define | **Uint128Low64**(x)   (x).first |
|  | |
| #define | **Uint128High64**(x)   (x).second |
|  | |

|  |  |
| --- | --- |
| Typedefs | |
| typedef uint8\_t | **uint8** |
|  | |
| typedef uint16\_t | **uint16** |
|  | |
| typedef uint32\_t | **uint32** |
|  | |
| typedef uint64\_t | **uint64** |
|  | |
| typedef struct \_uint128 | **uint128** |
|  | |

|  |  |
| --- | --- |
| Functions | |
| uint64\_t | **CityHash64** (const char \*buf, size\_t len) |
|  | |
| uint64\_t | **CityHash64WithSeed** (const char \*buf, size\_t len, uint64\_t seed) |
|  | |
| uint64\_t | **CityHash64WithSeeds** (const char \*buf, size\_t len, uint64\_t seed0, uint64\_t seed1) |
|  | |
| uint128 | **CityHash128** (const char \*s, size\_t len) |
|  | |
| uint128 | **CityHash128WithSeed** (const char \*s, size\_t len, uint128 seed) |
|  | |
| uint32 | **CityHash32** (const char \*buf, size\_t len) |
|  | |
| static uint64\_t | **Hash128to64** (const uint128 x) |
|  | |

## Detailed Description

functions for hashin strings, C translation of cityhash (C++, google)

Author
:   bdnt

See also
:   https://github.com/bdnt/cityhash-c
:   https://github.com/google/cityhash


---

Generated on Mon Mar 19 2018 23:42:01 for FastqPuri by  

 1.8.14
