# Supplementary material for: FastqPuri: high-performance preprocessing of RNA-seq data
Source: BMC Bioinformatics. 2019 May 3;20:226. doi: 10.1186/s12859-019-2799-0 (PMC6500068; doi:10.1186/s12859-019-2799-0)
Supplement: Supplementary file 2 — Archive of FastqPuri. Archive containing all files needed to install and run FastqPuri v1.0.6. Date stamp March 22, 2019. (GZ 47,819 kb) [file 12859_2019_2799_MOESM2_ESM.gz › FastqPuri-1.0.6/html/citycrc_8h.html]

FastqPuri: include/citycrc.h File Reference


|  |
| --- |
| FastqPuri |


- include

Functions

citycrc.h File Reference

functions for hashin strings, C translation of cityhash (C++, google)
More...

`#include "city.h"`

Include dependency graph for citycrc.h:

Go to the source code of this file.

|  |  |
| --- | --- |
| Functions | |
| uint128 | **CityHashCrc128** (const char \*s, size\_t len) |
|  | |
| uint128 | **CityHashCrc128WithSeed** (const char \*s, size\_t len, uint128 seed) |
|  | |
| void | **CityHashCrc256** (const char \*s, size\_t len, uint64 \*result) |
|  | |

## Detailed Description

functions for hashin strings, C translation of cityhash (C++, google)

Author
:   bdnt

See also
:   https://github.com/bdnt/cityhash-c
:   https://github.com/google/cityhash


---

Generated on Mon Mar 19 2018 23:42:01 for FastqPuri by  

 1.8.14
