# Supplementary material for: FastqPuri: high-performance preprocessing of RNA-seq data
Source: BMC Bioinformatics. 2019 May 3;20:226. doi: 10.1186/s12859-019-2799-0 (PMC6500068; doi:10.1186/s12859-019-2799-0)
Supplement: Supplementary file 2 — Archive of FastqPuri. Archive containing all files needed to install and run FastqPuri v1.0.6. Date stamp March 22, 2019. (GZ 47,819 kb) [file 12859_2019_2799_MOESM2_ESM.gz › FastqPuri-1.0.6/html/classes.html]

FastqPuri: Class Index


|  |
| --- |
| FastqPuri |


Class Index

\_ | s

|  |  |  |  |  |
| --- | --- | --- | --- | --- |
| |  | | --- | | \_ | | \_bfkmer | \_iparam\_makeBloom | \_node | \_uint128 |
|  | \_ds\_adap | \_iparam\_makeTree | \_split | \|  \| \| --- \| \| s \| |
| \_ad\_seq | \_fa\_data | \_iparam\_Qreport | \_stats\_TF |  |
| \_adapter | \_fa\_entry | \_iparam\_Sreport | \_stats\_TFDS | statsinfo |
| \_bfilter | \_fq\_read | \_iparam\_trimFilter | \_tree |  |
|  |  |  |  |  |

\_ | s


---

Generated on Mon Mar 19 2018 23:42:01 for FastqPuri by  

 1.8.14
