# Supplementary material for: FastqPuri: high-performance preprocessing of RNA-seq data
Source: BMC Bioinformatics. 2019 May 3;20:226. doi: 10.1186/s12859-019-2799-0 (PMC6500068; doi:10.1186/s12859-019-2799-0)
Supplement: Supplementary file 2 — Archive of FastqPuri. Archive containing all files needed to install and run FastqPuri v1.0.6. Date stamp March 22, 2019. (GZ 47,819 kb) [file 12859_2019_2799_MOESM2_ESM.gz › FastqPuri-1.0.6/html/config_8h_source.html]

My Project: config.h Source File


|  |
| --- |
| My Project |


config.h

1 #define VERSION "1.0"

2 #define HAVE\_RPKG

3 #define RSCRIPT\_EXEC "/usr/local/bin/compdiag/Rscript\_RBioC"

4 #define READ\_MAXLEN 400

5 #define RMD\_QUALITY\_REPORT "/home/loc03475/bighome/projects/FastqPuri/R/quality\_report.Rmd"

6 #define RMD\_SUMMARY\_REPORT "/home/loc03475/bighome/projects/FastqPuri/R/summary\_report.Rmd"

7 #define RMD\_SUMMARY\_FILTER\_REPORT "/home/loc03475/bighome/projects/FastqPuri/R/summary\_filter\_report.Rmd"

8 #define RMD\_SUMMARY\_FILTER\_REPORTDS "/home/loc03475/bighome/projects/FastqPuri/R/summary\_filter\_reportDS.Rmd"


---

Generated by  

 1.8.14
