# Supplementary material for: FastqPuri: high-performance preprocessing of RNA-seq data
Source: BMC Bioinformatics. 2019 May 3;20:226. doi: 10.1186/s12859-019-2799-0 (PMC6500068; doi:10.1186/s12859-019-2799-0)
Supplement: Supplementary file 2 — Archive of FastqPuri. Archive containing all files needed to install and run FastqPuri v1.0.6. Date stamp March 22, 2019. (GZ 47,819 kb) [file 12859_2019_2799_MOESM2_ESM.gz › FastqPuri-1.0.6/html/defines_8h.html]

FastqPuri: include/defines.h File Reference


|  |
| --- |
| FastqPuri |


- include

Macros

defines.h File Reference

Macro definitions.
More...

`#include <stdint.h>`  
`#include <inttypes.h>`

Include dependency graph for defines.h:

This graph shows which files directly or indirectly include this file:

Go to the source code of this file.

|  |  |
| --- | --- |
| Macros | |
| #define | B\_LEN   131072 |
|  | |
| #define | MAX\_FILENAME   300 |
|  | |
| #define | bool   int16\_t |
|  | |
| #define | true   1 |
|  | |
| #define | false   0 |
|  | |
| #define | max(a, b)   (((a) > (b)) ? (a) : (b)) |
|  | |
| #define | min(a, b)   (((a) < (b)) ? (a) : (b)) |
|  | |
| #define | mem\_usageMB() |
|  | |
| #define | mem\_usage() |
|  | |
| #define | DEFAULT\_MINQ   27 |
|  | |
| #define | DEFAULT\_NTILES   96 |
|  | |
| #define | DEFAULT\_NQ   46 |
|  | |
| #define | ZEROQ   33 |
|  | |
| #define | N\_ACGT   5 |
|  | |
| #define | MAX\_RCOMMAND   4000 |
|  | |
| #define | FA\_ENTRY\_BUF   20 |
|  | |
| #define | LOG\_4   0.60206 |
|  | |
| #define | MIN\_NMATCHES   12 |
|  | |
| #define | T\_ACGT   4 |
|  | |
| #define | NPOOL\_1D   1048576 |
|  | |
| #define | NPOOL\_2D   16 |
|  | |
| #define | MAX\_FASZ\_TREE   1e7 |
|  | |
| #define | BITSPERCHAR   8 |
|  | |
| #define | BASESPERCHAR   4 |
|  | |
| #define | KMER\_LEN   25 |
|  | |
| #define | FALSE\_POS\_RATE   0.05 |
|  | |
| #define | ZERO\_POS\_RATE   1e-14 |
|  | |
| #define | NO   0 |
|  | |
| #define | ALL   1 |
|  | |
| #define | ENDS   2 |
|  | |
| #define | STRIP   3 |
|  | |
| #define | FRAC   3 |
|  | |
| #define | ENDSFRAC   4 |
|  | |
| #define | GLOBAL   5 |
|  | |
| #define | TREE   1 |
|  | |
| #define | BLOOM   2 |
|  | |
| #define | ERROR   1000 |
|  | |
| #define | DEFAULT\_MINL   25 |
|  | |
| #define | ADAP   0 |
|  | |
| #define | CONT   1 |
|  | |
| #define | LOWQ   2 |
|  | |
| #define | NNNN   3 |
|  | |
| #define | GOOD   4 |
|  | |
| #define | NFILTERS   4 |
|  | |
| #define | ADAP2   5 |
|  | |
| #define | CONT2   6 |
|  | |
| #define | LOWQ2   7 |
|  | |
| #define | NNNN2   8 |
|  | |
| #define | GOOD2   9 |
|  | |
| #define | NFILES\_DS   10 |
|  | |

## Detailed Description

Macro definitions.

Author
:   Paula Perez paula.nosp@m.pere.nosp@m.zrubi.nosp@m.o@gm.nosp@m.ail.c.nosp@m.om

Date
:   07.08.2017

## Macro Definition Documentation

## ◆ ADAP

|  |
| --- |
| #define ADAP   0 |

Adapter filter

## ◆ ADAP2

|  |
| --- |
| #define ADAP2   5 |

Adapter filter read2

## ◆ ALL

|  |
| --- |
| #define ALL   1 |

Trims if a lowQ base calling | N is found

## ◆ B\_LEN

|  |
| --- |
| #define B\_LEN   131072 |

buffer size

## ◆ BASESPERCHAR

|  |
| --- |
| #define BASESPERCHAR   4 |

number of nucleotides that can fit in a char

## ◆ BITSPERCHAR

|  |
| --- |
| #define BITSPERCHAR   8 |

number of bits in a char

## ◆ BLOOM

|  |
| --- |
| #define BLOOM   2 |

Use a bloom filter to look for contaminations

## ◆ bool

|  |
| --- |
| #define bool   int16\_t |

define a bool type

## ◆ CONT

|  |
| --- |
| #define CONT   1 |

Contamination filter

## ◆ CONT2

|  |
| --- |
| #define CONT2   6 |

Contamination filter read2

## ◆ DEFAULT\_MINL

|  |
| --- |
| #define DEFAULT\_MINL   25 |

Default minimum length under which we discard the reads

## ◆ DEFAULT\_MINQ

|  |
| --- |
| #define DEFAULT\_MINQ   27 |

Minimum quality threshold

## ◆ DEFAULT\_NQ

|  |
| --- |
| #define DEFAULT\_NQ   46 |

Default number of different quality values

## ◆ DEFAULT\_NTILES

|  |
| --- |
| #define DEFAULT\_NTILES   96 |

Default number of tiles

## ◆ ENDS

|  |
| --- |
| #define ENDS   2 |

Trims at the ends

## ◆ ENDSFRAC

|  |
| --- |
| #define ENDSFRAC   4 |

trims at the ends and discards a read if the remaining part has more than > percent lowQ bases

## ◆ ERROR

|  |
| --- |
| #define ERROR   1000 |

Encodes an error when reading in trimN, trimQ, method options in trimFilter

## ◆ FA\_ENTRY\_BUF

|  |
| --- |
| #define FA\_ENTRY\_BUF   20 |

buffer for fasta entries

## ◆ false

|  |
| --- |
| #define false   0 |

assign false to 0

## ◆ FALSE\_POS\_RATE

|  |
| --- |
| #define FALSE\_POS\_RATE   0.05 |

default false positive rate

## ◆ FRAC

|  |
| --- |
| #define FRAC   3 |

Discards a read if it contains > percent lowQ bases

## ◆ GLOBAL

|  |
| --- |
| #define GLOBAL   5 |

Trims a fixed # bases from e left and right

## ◆ GOOD

|  |
| --- |
| #define GOOD   4 |

Good reads

## ◆ GOOD2

|  |
| --- |
| #define GOOD2   9 |

Good reads read2

## ◆ KMER\_LEN

|  |
| --- |
| #define KMER\_LEN   25 |

default kmer length

## ◆ LOG\_4

|  |
| --- |
| #define LOG\_4   0.60206 |

log\_10(4) for the adapters alignment score

## ◆ LOWQ

|  |
| --- |
| #define LOWQ   2 |

Low quality filter

## ◆ LOWQ2

|  |
| --- |
| #define LOWQ2   7 |

Low quality filter read2

## ◆ max

|  |  |  |  |
| --- | --- | --- | --- |
| #define max | ( |  | a, |
|  |  |  | b |
|  | ) |  | (((a) > (b)) ? (a) : (b)) |

max function

## ◆ MAX\_FASZ\_TREE

|  |
| --- |
| #define MAX\_FASZ\_TREE   1e7 |

Maximum fasta size for constructing a tree. DECIDE A SENSIBLE SIZE

## ◆ MAX\_FILENAME

|  |
| --- |
| #define MAX\_FILENAME   300 |

Maximum # chars in a filename

## ◆ MAX\_RCOMMAND

|  |
| --- |
| #define MAX\_RCOMMAND   4000 |

Maximum # chars in R command

## ◆ mem\_usage

|  |  |  |  |  |
| --- | --- | --- | --- | --- |
| #define mem\_usage | ( |  | ) |  |

**Value:**

fprintf(stderr, \

"- Current allocated memory: %" PRIu64 "Bytes.\n", \

alloc\_mem)

alloc\_mem

uint64\_t alloc\_mem

**Definition:** makeBloom.c:42

returns allocated memory in Bytes

## ◆ mem\_usageMB

|  |  |  |  |  |
| --- | --- | --- | --- | --- |
| #define mem\_usageMB | ( |  | ) |  |

**Value:**

fprintf(stderr, \

"- Current allocated memory: %" PRIu64 "MB.\n", \

alloc\_mem >> 20)

alloc\_mem

uint64\_t alloc\_mem

**Definition:** makeBloom.c:42

returns allocated memory in MB

## ◆ min

|  |  |  |  |
| --- | --- | --- | --- |
| #define min | ( |  | a, |
|  |  |  | b |
|  | ) |  | (((a) < (b)) ? (a) : (b)) |

min function

## ◆ MIN\_NMATCHES

|  |
| --- |
| #define MIN\_NMATCHES   12 |

minimum number of matches demanded

## ◆ N\_ACGT

|  |
| --- |
| #define N\_ACGT   5 |

Number of different nucleotides in the fq file

## ◆ NFILES\_DS

|  |
| --- |
| #define NFILES\_DS   10 |

number of outputfiles in double stranded case

## ◆ NFILTERS

|  |
| --- |
| #define NFILTERS   4 |

total number of filters

## ◆ NNNN

|  |
| --- |
| #define NNNN   3 |

N's presence filter

## ◆ NNNN2

|  |
| --- |
| #define NNNN2   8 |

N's presence filter read2

## ◆ NO

|  |
| --- |
| #define NO   0 |

No trimming

## ◆ NPOOL\_1D

|  |
| --- |
| #define NPOOL\_1D   1048576 |

Number of Node structs allocated in inner dim

## ◆ NPOOL\_2D

|  |
| --- |
| #define NPOOL\_2D   16 |

Number of \*Node allocated in outer dim

## ◆ STRIP

|  |
| --- |
| #define STRIP   3 |

Looks for the largest N-free sequence

## ◆ T\_ACGT

|  |
| --- |
| #define T\_ACGT   4 |

Number of children per node in tree

## ◆ TREE

|  |
| --- |
| #define TREE   1 |

Use a tree to look for contaminations

## ◆ true

|  |
| --- |
| #define true   1 |

assign true to 1

## ◆ ZERO\_POS\_RATE

|  |
| --- |
| #define ZERO\_POS\_RATE   1e-14 |

0 threshold for a double

## ◆ ZEROQ

|  |
| --- |
| #define ZEROQ   33 |

ASCII code of lowest quality value (!)


---

Generated on Mon Mar 19 2018 23:42:01 for FastqPuri by  

 1.8.14
