# Supplementary material for: FastqPuri: high-performance preprocessing of RNA-seq data
Source: BMC Bioinformatics. 2019 May 3;20:226. doi: 10.1186/s12859-019-2799-0 (PMC6500068; doi:10.1186/s12859-019-2799-0)
Supplement: Supplementary file 2 — Archive of FastqPuri. Archive containing all files needed to install and run FastqPuri v1.0.6. Date stamp March 22, 2019. (GZ 47,819 kb) [file 12859_2019_2799_MOESM2_ESM.gz › FastqPuri-1.0.6/html/dir_d44c64559bbebec7f509842c48db8b23.html]

FastqPuri: include Directory Reference


|  |
| --- |
| FastqPuri |


- include

include Directory Reference

|  |  |
| --- | --- |
| Files | |
| file | adapters.h [code] |
|  | sequence manipulation for alignment |
|  | |
| file | bloom.h [code] |
|  | functions that implement the bloom filter |
|  | |
| file | city.h [code] |
|  | functions for hashin strings, C translation of cityhash (C++, google) |
|  | |
| file | citycrc.h [code] |
|  | functions for hashin strings, C translation of cityhash (C++, google) |
|  | |
| file | defines.h [code] |
|  | Macro definitions. |
|  | |
| file | fa\_read.h [code] |
|  | reads in and stores fasta files |
|  | |
| file | fopen\_gen.h [code] |
|  | Uncompress/compress input/output files using pipes. |
|  | |
| file | fq\_read.h [code] |
|  | fastq entries manipulations (read/write) |
|  | |
| file | init\_makeBloom.h [code] |
|  | Help dialog for makeBloom and initialization of the command line arguments. |
|  | |
| file | init\_makeTree.h [code] |
|  | Help dialog for makeTree and initialization of the command line arguments. |
|  | |
| file | init\_Qreport.h [code] |
|  | Header file: help dialog for Qreport and initialization of the command line arguments. |
|  | |
| file | init\_Sreport.h [code] |
|  | Help dialog for Sreport and initialization of the command line arguments. |
|  | |
| file | init\_trimFilter.h [code] |
|  | help dialog for trimFilter and initialization of the command line arguments. |
|  | |
| file | init\_trimFilterDS.h [code] |
|  | help dialog for trimFilterDS and initialization of the command line arguments. |
|  | |
| file | io\_trimFilter.h [code] |
|  | buffer fq output, write summary file |
|  | |
| file | io\_trimFilterDS.h [code] |
|  | buffer fq output, write summary file |
|  | |
| file | Lmer.h [code] |
|  | Manipulation of Lmers and sequences. |
|  | |
| file | Rcommand\_Qreport.h [code] |
|  | get Rscript command for Qreport |
|  | |
| file | Rcommand\_Sreport.h [code] |
|  | get Rscript command for Sreport |
|  | |
| file | stats\_info.h [code] |
|  | Construct the quality report variables and update them. |
|  | |
| file | str\_manip.h [code] |
|  | functions that do string manipulation |
|  | |
| file | struct\_trimFilter.h [code] |
|  | structure where the input arguments of trimFilter and trimFilterDS will be stored and function to free the memory of it. |
|  | |
| file | tree.h [code] |
|  | Construction of tree, check paths, write tree, read in tree. |
|  | |
| file | trim.h [code] |
|  | trims/filter sequences after Quality, N's contaminations. |
|  | |
| file | trimDS.h [code] |
|  | trim adapters from double stranded data |
|  | |


---

Generated on Mon Mar 19 2018 23:42:01 for FastqPuri by  

 1.8.14
