# Supplementary material for: FastqPuri: high-performance preprocessing of RNA-seq data
Source: BMC Bioinformatics. 2019 May 3;20:226. doi: 10.1186/s12859-019-2799-0 (PMC6500068; doi:10.1186/s12859-019-2799-0)
Supplement: Supplementary file 2 — Archive of FastqPuri. Archive containing all files needed to install and run FastqPuri v1.0.6. Date stamp March 22, 2019. (GZ 47,819 kb) [file 12859_2019_2799_MOESM2_ESM.gz › FastqPuri-1.0.6/html/fa__read_8h.html]

FastqPuri: include/fa\_read.h File Reference


|  |
| --- |
| FastqPuri |


- include

Classes |
Typedefs |
Functions

fa\_read.h File Reference

reads in and stores fasta files
More...

`#include <stdint.h>`

Include dependency graph for fa\_read.h:

This graph shows which files directly or indirectly include this file:

Go to the source code of this file.

|  |  |
| --- | --- |
| Classes | |
| struct | \_fa\_entry |
|  | fasta entry More... |
|  | |
| struct | \_fa\_data |
|  | stores sequences of a fasta file More... |
|  | |

|  |  |
| --- | --- |
| Typedefs | |
| typedef struct \_fa\_entry | Fa\_entry |
|  | fasta entry |
|  | |
| typedef struct \_fa\_data | Fa\_data |
|  | stores sequences of a fasta file |
|  | |

|  |  |
| --- | --- |
| Functions | |
| int | read\_fasta (char \*filename, Fa\_data \*ptr\_fa) |
|  | reads a fasta file and stores the contents in a Fa\_data structure. More... |
|  | |
| uint64\_t | size\_fasta (Fa\_data \*ptr\_fa) |
|  | computes length of genome in fasta structure More... |
|  | |
| uint64\_t | nkmers (Fa\_data \*ptr\_fa, int kmersize) |
|  | number of kmers of length kmersize contained in a fasta structure More... |
|  | |
| void | free\_fasta (Fa\_data \*ptr\_fa) |
|  | free fasta file More... |
|  | |

## Detailed Description

reads in and stores fasta files

Author
:   Paula Perez paula.nosp@m.pere.nosp@m.zrubi.nosp@m.o@gm.nosp@m.ail.c.nosp@m.om

Date
:   16.08.2017

## Function Documentation

## ◆ free\_fasta()

|  |  |  |  |  |  |
| --- | --- | --- | --- | --- | --- |
| void free\_fasta | ( | Fa\_data \* | *ptr\_fa* | ) |  |

free fasta file

Parameters
:   |  |  |
    | --- | --- |
    | ptr\_fa | pointer to Fa\_data structure. |

The dynamically allocated memory in a Fa\_data struct is deallocated and counted, so that we can

## ◆ nkmers()

|  |  |  |  |
| --- | --- | --- | --- |
| uint64\_t nkmers | ( | Fa\_data \* | *ptr\_fa*, |
|  |  | int | *kmersize* |
|  | ) |  |  |

number of kmers of length kmersize contained in a fasta structure

Returns
:   number of kmers of length kmersize contained in a fasta structure

## ◆ read\_fasta()

|  |  |  |  |
| --- | --- | --- | --- |
| int read\_fasta | ( | char \* | *filename*, |
|  |  | Fa\_data \* | *ptr\_fa* |
|  | ) |  |  |

reads a fasta file and stores the contents in a Fa\_data structure.

Parameters
:   |  |  |
    | --- | --- |
    | filename | path to a fasta input file. |
    | ptr\_fa | pointer to Fa\_data structure. |

Returns
:   number of entries in the fasta file.

A fasta file is read and stored in a structure Fa\_data The basic problem with reading FASTA files is that there is no end-of-record indicator. When you're reading sequence n, you don't know you're done until you've read the header line for sequence n+1, which you won't parse 'til later (when you're reading in the sequence n+1). The solution implemented here is to read the file twice. The first time, (sweep\_fa), we initialize Fa\_data and store the parameters:

- nlines: number of lines of the fasta file.
- nentries: number of entries in the fasta file.
- linelen: length of a line in the considered fasta file.
- entrylen: array containing the lengths of every entry. With this information, the pointer to Fa\_entry can be allocated and the file is read again and the entries are stored in the structure.

## ◆ size\_fasta()

|  |  |  |  |  |  |
| --- | --- | --- | --- | --- | --- |
| uint64\_t size\_fasta | ( | Fa\_data \* | *ptr\_fa* | ) |  |

computes length of genome in fasta structure

Parameters
:   |  |  |
    | --- | --- |
    | ptr\_fa | pointer to Fa\_data |

Returns
:   total number of nucleotides


---

Generated on Mon Mar 19 2018 23:42:01 for FastqPuri by  

 1.8.14
